# Isolation of the symbiotic bacterium *Brevundimonas aurantiaca* and its potential role in modulating reproductive traits in *Culex pipiens pallens*

**DOI:** 10.1186/s13071-026-07575-y

**Published:** 2026-07-24

**Authors:** Xin-Mei Wang, Xiu-Xia Guo, Peng Cheng, Hai-Fang Wang, Gang Li, Hui Xu, Yu-Xiu Shang, Jing-Jing Lei, Mao-Qing Gong, Li-Juan Liu

**Affiliations:** 1 Shandong Institute of Parasitic Diseases, Shandong First Medical University and Shandong Academy of Medical Sciences, Jining, China; 2 School of Public Health, Shandong First Medical University and Shandong Academy of Medical Sciences, Jinan, China

**Keywords:** *Culex pipiens pallens*, Autogeny, Symbiotic bacteria, *Brevundimonas aurantiaca*

## Abstract

**Background:**

*Culex pipiens pallens* is a significant vector of mosquito-borne diseases, and its autogenous trait exerts a critical impact on population expansion and disease transmission risks. Understanding the regulatory effect of gut-specific symbiotic bacteria on the autogeny of *Cx. pipiens pallens* will provide a microbial perspective to elucidate the mechanisms underlying the formation of autogenous reproductive strategies and provide a theoretical basis for the development of novel mosquito-borne disease control.

**Methods:**

Autogenous *Cx. pipiens pallens* were utilized to isolate and culture gut-specific symbiotic bacteria. Green fluorescent protein (GFP) labeling technology and absolute quantitative real-time polymerase chain reaction (qRT-PCR) were used to confirm the colonization ability of the symbiotic bacteria in the guts of anautogenous *Cx. pipiens pallens*. The alterations in the expression levels of reproductive-related genes were verified by relative qRT-PCR. 20-Hydroxyecdysone (20E), juvenile hormone (JH), and the concentrations of vitellogenin were measured to assess the vitellogenesis. Continuous multi-generational treatment with bacteria was applied to anautogenous *Cx. pipiens pallens* to evaluate transgenerational effects. Larval stage, pupation rate, pupal stage, emergence rate, male:female ratio, blood-feeding rate, oviposition rate, and hatching rate were recorded to assess the effects of the symbiotic bacteria on the reproductive and developmental phenotypes in the anautogenous *Cx. pipiens pallens*.

**Results:**

The gut-specific symbiotic bacterium isolated from autogenous *Cx. pipiens pallens* was identified as *Brevundimonas aurantiaca*, a Gram-negative rod-shaped bacterium that forms orange-yellow circular colonies on *Caulobacter* medium. *B. aurantiaca* successfully colonized in the midgut of anautogenous *Cx. pipiens pallens* and achieved vertical transmission. The reproductive phenotype of anautogenous *Cx. pipiens pallens* is complex and significant alterations were observed in two aspects investigated. By the fifth generation, the bacteria-treated group compared with the untreated anautogenous group showed decreased blood-feeding rate (87.7% vs 96.3%) and prolonged larval development (8.3 d vs 7.4 d).

**Conclusions:**

The symbiotic bacterium *B. aurantiaca* may be associated with changes in the reproductive strategy of anautogenous *Cx. pipiens pallens*, although direct mechanistic evidence is not provided in this study. These findings not only provide an important microbial regulatory perspective for understanding the molecular mechanisms underlying autogeny in mosquitoes, but also offer potential targets for the development of novel symbiont-based biocontrol strategies against mosquito-borne diseases.

**Graphical Abstract:**

**Figure:**
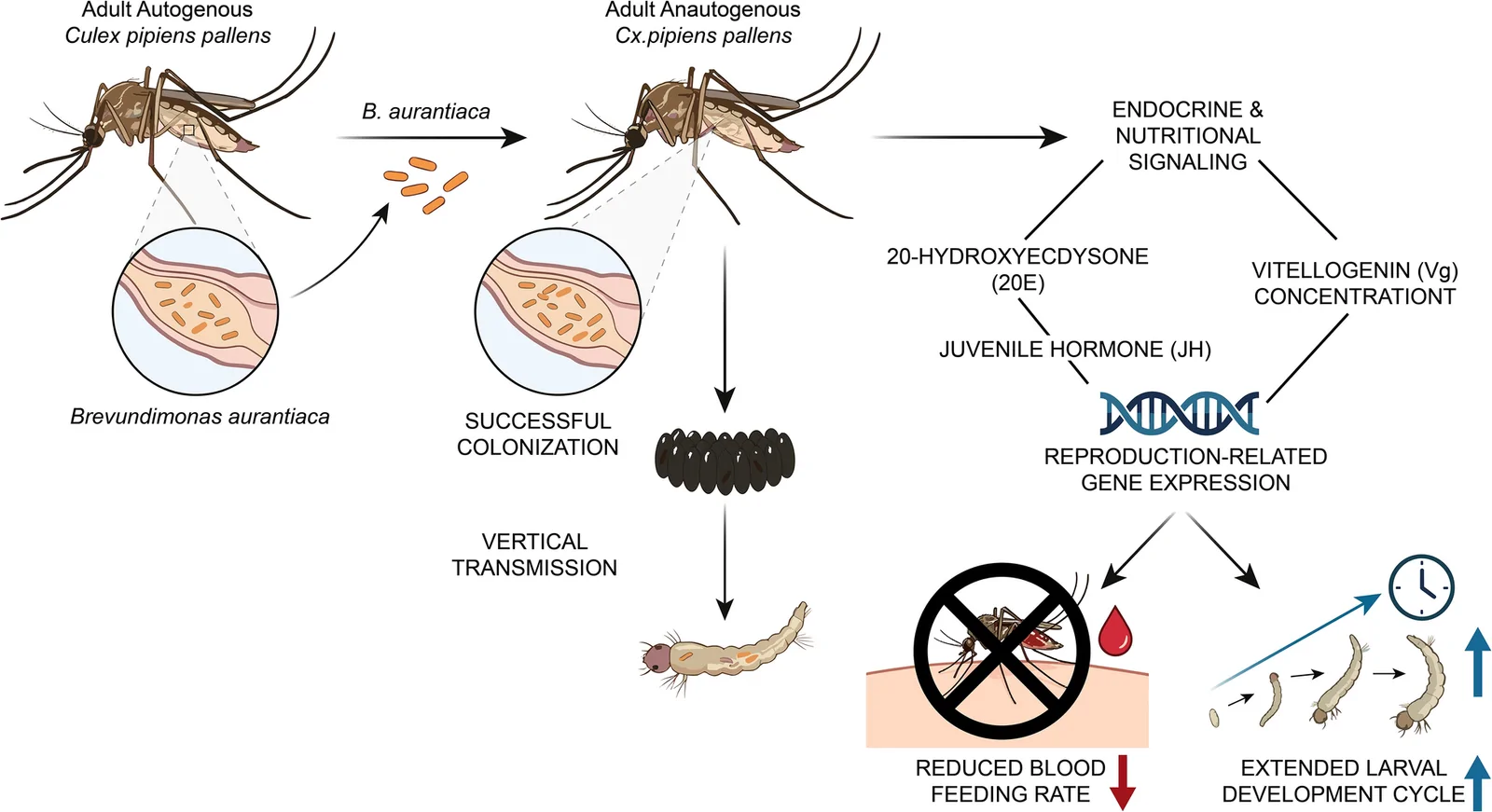

**Supplementary Information:**

The online version contains supplementary material available at https://doi.org/10.1186/s13071-026-07575-y.

## Background

*Culex pipiens pallens* (Diptera: Culicidae) is the dominant mosquito species in Northern China. It also serves as a major vector for various pathogens including Japanese encephalitis virus, Bancroftian filaria, and West Nile virus. Fluctuations in its population directly influence the transmission risk and epidemic intensity of mosquito-borne diseases [1, 2]. Blood-feeding by mosquitoes facilitates viral transmission among hosts, thus triggering the outbreak and prevalence of mosquito-borne diseases. Additionally, the reproductive strategies of mosquitoes are closely associated with their disease transmission capacity. Female mosquitoes normally require a blood meal to acquire protein nutrients essential for ovarian development, but some mosquito species or populations possess an autogenous trait, which allows these female mosquitoes to complete ovarian development and oviposition without blood-feeding [3]. Differences in the autogenous and anautogenous reproductive strategies not only relate to the ecological adaptability of mosquito populations but also provide an ideal biological model for investigating the regulatory mechanisms underlying mosquito reproductive development [4]. Currently, mosquito species showing autogenous behavior are known to span 15 genera and 68 species [5], including *Aedes albopictus* [6], *Culiseta longiareolata* [7], *Ae. atropalpu*s [8], *Ae. caspius* [9], and *Cx. pipiens molestus* [10]. Autogeny in mosquitoes not only facilitates the rapid establishment and persistence of populations and reduces their dependence on exogenous blood sources but may also decrease the proportion of pathogen infected individuals within populations, thus conferring significant ecological and epidemiological implications [11–13]. Although autogeny has been demonstrated to be regulated by genetic, nutritional, and hormonal factors [8, 14, 15], its underlying molecular mechanisms remain incompletely understood.

In recent years, research on the symbiotic relationships between insects and microorganisms has advanced considerably, and the gut microbiota have been found to be extensively involved in key biological processes of the host, including growth and development, nutrient metabolism, immune defense, and reproductive regulation [14, 16–19]. Specific gut microbiota have been reported to mediate the reproductive capacity of mosquitoes by influencing nutrient supply, hormone synthesis, and signaling pathways [20]. For example, *Rahnella aquatilis* impedes ovarian development and fecundity in *Ae. albopictus* by decreasing the levels of ecdysone and vitellogenin in the mosquito [21]. In *Ae. aegypti*, certain gut bacteria capable of synthesizing vitamin B are essential for the normal reproductive function of the host [22]. Sterilization decreases the production of ecdysone and the synthesis of vitellogenin in the fat body of *Ae. aegypti*, leading to reduced egg production [23]. *Lysinibacillus sphaericus* downregulates *Vg* expression by inhibiting lysosomal function and the TOR signaling pathway, thereby substantially suppressing oviposition in female *Anopheles dirus* [24]. Together, these studies suggest that the gut microbiota may be one of the key factors driving the differentiation of mosquito reproductive strategies and their adaptive evolution.

Previously, by comparing the gut microbiota between autogenous and anautogenous strains of *Cx. pipiens pallens*, we isolated and cultured one dominant bacterial strain (*Aeromonas hydrophila*, relative abundance 26.1 ± 25.2%) and three unique strains (*Pseudomonas putida*, 5.5 ± 2.8%, *Brevundimonas aurantiaca*, 3.7 ± 1.9% and *Microbacterium maritypicum*, 4.4 ± 2.3%) from the guts of autogenous mosquitoes [25]. When anautogenous mosquitoes were individually fed with these isolated strains, the highest total yolk protein content was observed in the group treated with *B. aurantiaca* (data not shown). Therefore, we selected this strain for further study. We acknowledge that other gut symbionts may also contribute to the autogenous phenotype, however, focusing on *B. aurantiaca* as a starting point allows a more targeted investigation of its potential role. Furthermore, the symbiotic bacterial community in mosquitoes is a dynamic and unstable system, whereby newly acquired symbionts are often unable to stably colonize the host over long term and may be lost during subsequent developmental or reproductive processes [26–28]. A single-generation bacterial treatment may only induce a transient physiological response in the host. Hence, a multi-generational continuous treatment with *B. aurantiaca* is required to assess its transgenerational effects. Meanwhile, enzymatic assays revealed that *B. aurantiaca* produces neither digestive enzymes nor amylase. Combined with its association with increased yolk protein content in our preliminary experiment, we hypothesize that this bacterium may influence mosquito blood-feeding behavior, ovarian development, and reproductive output through alternative, non-digestive enzyme-mediated pathways, such as regulating host reproduction-related genes and reproductive physiological pathways. The present study aims to characterize the physiological and biochemical properties of *B. aurantiaca* and its colonization ability and autogeny effects in *Cx. pipiens pallens*, as well as to preliminarily elucidate its mechanism of action through analysis of reproduction-related gene expression levels and vitellogenesis assay. The findings of this study contribute to understanding the components of the autogenous phenotype, thereby providing a preliminary theoretical basis for the development of novel mosquito-borne disease control strategies based on microbial regulation.

### Methods

#### Test insect source

The *Cx. pipiens pallens* used in the experiments were provided by Jinan Center for Disease Control and Prevention in Shandong Province and had been routinely reared at Shandong Institute of Parasitic Diseases for 18 years. The autogenous strain was domesticated from this parental strain in the laboratory and has been maintained for 36 consecutive generations to date. Mosquitoes were reared under the following standard conditions: temperature of 25 ± 2 °C, relative humidity of 60.0% ± 5.0%, and a photoperiod of 12 h light:12 h dark. Larvae were fed a mixture of homemade pork liver powder and yeast powder (Shandong Lukang Pharmaceutical Co., LTD) at a ratio of 1:3(w/w). The feed was suspended in distilled water at a concentration of 0.5 g/L. Before each feeding, the surface film (oil film) on the rearing water was gently removed by sweeping a sterile fine-mesh screen across the water surface. The daily amount of feed suspension added to each rearing tray was determined based on the consumption rate of the larvae in the experimental group on the previous day; the control group received an identical volume of fresh suspension. The suspension was replenished twice daily (morning and evening) to ensure ad libitum access while maintaining equal total feeding volumes between groups. Adult mosquitoes were provided with a 10.0% glucose solution. The glucose solution was provided ad libitum and replaced daily with freshly prepared sterile 10.0% glucose to ensure consistent nutrient availability and prevent microbial contamination.

### Isolation and identification of the symbiotic bacterium *B. aurantiaca*

Autogenous adult female mosquitoes of *Cx. pipiens pallens* were collected and divided into groups of 10, with three biological replicates per group. After being anesthetized with CO_2_, mosquitoes were surface sterilized with 75.0% ethanol followed by three washes with sterile phosphate-buffered saline (PBS) to minimize potential surface nucleic acid contamination [18]. The midgut tissue was harvested by aseptic dissection and placed in a sterile 1.5-mL centrifuge tube. Then, 500 μL of 0.9% saline solution was added, and the sample was thoroughly homogenized. The homogenate was sequentially at 10^–4^ and 10^–5^ concentrations, and then 100 μL of each dilution was spread evenly onto a 9-cm-diameter *Caulobacter* medium (CM) agar plate (Bacto peptone, 2.0 g/L; Yeast extract, 1.0 g/L; MgSO₄·7H₂O, 0.2 g/L; Agar, 15.0 g/L) [29]. The plate was inverted and incubated at 30 °C in a constant-temperature incubator for 24 h. Based on characteristics such as colony size, morphology, and color, a single colony was picked and streaked into three zones on fresh CM medium for purification, continuing until a morphologically uniform single colony was obtained. Purified colonies were inoculated into 15-mL centrifuge tubes containing 3 mL of CM liquid medium and incubated by shaking at 30 °C and 180 rpm for 24 h to obtain the bacterial culture. The bacterial culture samples were sent to Sangon Biotechnology Co., Ltd. (Shanghai, China) for 16S rRNA gene sequencing. The resulting sequences were subjected to BLAST alignment in the National Center for Biotechnology Information (NCBI) database (https://www.ncbi.nlm.nih.gov). Using sequences from reference strains with ≥ 97.0% homology as templates, a phylogenetic tree was created utilizing the Neighbor-Joining (NJ) method with MEGA 11.0 software. Bootstrap analysis was conducted with 1000 repetitions to evaluate tree reliability [30]. The taxonomic status of the strains was determined by integrating sequence homology alignment results with the phylogenetic tree analysis. After the bacteria were treated according to previous methods [31], the morphology of the bacteria was observed by scanning electron microscopy. The parental strain of *B. aurantiaca* used in this study was isolated from autogenous *Cx. pipiens pallens* as described above. The strain was stored at −80 °C in 20% glycerol. Prior to each experiment, the strain was reactivated on CM agar plates, and the third generation of subculture was used for all subsequent assays to ensure physiological consistency.

### Determination of *B. aurantiaca* growth curve and physiological and biochemical tests

The growth curve of the strains was determined by spectrophotometry. A single colony of *B. aurantiaca* was inoculated into 5 mL of CM liquid medium, followed by shaking incubation at 180 rpm in a 30 °C incubator for 16 h to obtain a seed culture in the logarithmic growth phase. The seed culture was then transferred into Erlenmeyer flasks containing fresh CM liquid medium at an inoculum size of 2.0% (v/v), with three biological replicates set for each group. A tube with uninoculated medium served as the blank control. The inoculated cultures were incubated continuously in a constant temperature shaking incubator at 30 °C and 180 rpm for 48 h. The samples were collected at 1 h intervals, starting from the initial incubation time (0 h), and the absorbance value at a wavelength of 600 nm (OD600) was measured utilizing a microplate reader.

The physiological and biochemical characteristics of this strain were determined using the API 20NE test strips (bioMérieux) for the identification of non-fastidious, non-enteric Gram-negative bacilli, with the experimental procedures performed strictly in accordance with the manufacturer’s instructions.

### Construction of GFP-labeled bacteria and its colonization in the gut of anautogenous *Cx. pipiens pallens*

To track the colonization of *B. aurantiaca* in the intestinal tract of anautogenous *Cx. pipiens pallens*, the target strain *B. aurantiaca* isolated and identified in this study was employed as the host strain. A fluorescent expression plasmid carrying the green fluorescent protein (sfGFP)-coding gene and kanamycin resistance (50 μg/mL) screening marker was constructed and termed pET28(+) -sfGFP (General Biol (Anhui, China) Co., Ltd‌). Competent cells of *B. aurantiaca* were prepared via a modified rubidium chloride method following the instructions of the Ultra-Competent Cell Preps Kit (Sangon Biotech, Shanghai, China). The fluorescent plasmid pET28(+)-sfGFP was then transformed into the competent cells. Positive transformants were inoculated into CM liquid medium supplemented with 50 μg/mL kanamycin and cultured with shaking at 180 rpm and 30 °C for 12 h to yield a large quantity of sfGFP-expressing bacteria. The culture was centrifuged at 6000 rpm (approximately 4829 g) for 10 min to pellet the bacteria. The pellet was then washed three times with sterile PBS and mixed with a 10.0% sucrose solution at a 1:9 ratio to an approximate final concentration of 3.6 × 10^9^ colony- forming units (CFU)/mL to produce the bacterial–sucrose mixture feeding solution [32]. Newly emerged female mosquitoes were selected for the experiment. Mosquitoes in the experimental group were continuously fed the aforementioned bacteria–sucrose suspension for 3 consecutive days with fresh suspensions replaced daily, and then were separated into two groups and fed either blood or sterilized 10.0% glucose solution for 3 days. Mosquitoes in the control group were fed with a sterile 10.0% glucose solution throughout the entire experimental period, with a subgroup offered a blood meal on the third day. Egg rafts oviposited following blood feeding were transferred to sterile water and hatched larvae were reared to the second instar. On the third day after the switch to the sterile glucose solution, the midguts of mosquitoes in each group were dissected and placed on glass slides with 10 μL of sterile PBS added. Green fluorescence was observed utilizing the BioTek Cytation 1 Full-Automatic Imaging System (BioTek, USA) and Gen5 CHS 3.05 software.

### qRT-PCR assays for the absolute quantification of *B. aurantiaca* in mosquito samples

Egg rafts, larvae, pupae, and newly emerged female adults of autogenous *Cx. pipiens pallens* were selected for analysis. The bacterial load was determined in one-day-old emerged female of the first generation from the anautogenous *Cx. pipiens pallens* treated with *B. aurantiaca* (the bacterial treatment was performed as described previously). Each biological replicate consisted of one egg raft (approximately 100–150 eggs), 10 adult female mosquitoes, 10 second-instar larvae, and 10 pupae. Three biological replicates were performed per developmental stage. Total DNA was isolated from all samples utilizing the SteadyPure Universal Genomic DNA Extraction Kit (Accurate Biotechnology (Hunan, China) Co., Ltd.). The abundance of *B. aurantiaca* was quantified via absolute quantitative real-time polymerase chain reaction (qRT-PCR). Primers were designed for the strain-specific gene *crtW* (GenBank ID: AY166610.2) encoding β-carotene ketolase (β-carotene 4, 4’-oxygenase), a key enzyme in the carotenoid biosynthesis pathway that introduces keto groups at the 4 and 4’ positions of the β-ionone rings (Table 1) [33], and the amplified fragment was inserted into the pUC56 plasmid to construct a standard template. The standard template was subjected to tenfold serial dilution to produce a concentration gradient ranging from 3.29 × 10^8^ to 3.29 × 10^1^ copies. qRT-PCR reactions were conducted in a 20-μL reaction system utilizing the serially diluted standard template as the template, with the reaction conditions as follows: initial denaturation at 95 °C for 180 s; 50 cycles of denaturation at 95 °C for 10 s and annealing/extension at 60 °C for 30 s; and a final melting curve at 95 °C for 10 s, 65 °C for 60 s, and 97 °C for 1 s. A standard curve was graphed with the logarithm of the copy number of the standard template as the abscissa and the corresponding cycle threshold (Ct) value as the ordinate. Sample DNA was utilized as the template for the qRT-PCR reactions, which was performed in parallel with the plasmid standard template for standard curve construction. After obtaining the Ct values of the samples, the log10 copy number was calculated according to the standard curve to determine the abundance of *B. aurantiaca* in each sample.

**Table 1:** qRT-PCR primers used to measure bacterial colonization and reproductive gene expression

Primer name	Forward primer (5′-3′)		Reverse primer (5′-3′)
*crtW*	CCTGCTCACCTGTTTCCACTTCG	CCAGCCCTGAATCAAGACTCG	
*β-actin*	AGCGTGAACTGACGGCTCTTG	ACTCGTCGTACTCCTGCTTGG	
*Vg1*	TTCCCACCGTATTTTCCCCC	CGATAGCAAGGCCATAGAACAAAG	
*Vg3*	CCAATCCGTGCTTCAACGAG	GATCGAGTTTAGGCCATTGGG	
*VgR*	CTGTGATGCGGTGAATGGGA	TTGCCTTCGTCTTTGCCATC	
*Met* [34]	AACCAAAACTACCCGCCACCTC	CATCTGATGCCCATAACTGACCC	
*EcR*	TCTATTCAAGTACGCGCTCCT	ACAAATCTGGCAACACTTCGG	
*ILPR*	CGGCTTACACCTTGGTAATGACA	AAATACATGCTTCCCGCCAAC	

### qRT-PCR assays for the relative quantification of reproduction-related genes in females and the vitellogenesis assay

To investigate the initial effects of *B. aurantiaca* on the ovarian development of anautogenous female mosquitoes, an experimental group (FZYB group) was created. Newly emerged female mosquitoes were fed with a glucose solution supplemented with a bacterial culture (bacterial treatment as previously mentioned). A control group (FZY group) was established by feeding newly emerged female mosquitoes with a 10.0% sterile glucose solution. Cotton balls soaked in the glucose solution were replaced daily to ensure continuous supply. Female mosquito samples were collected at various time points. After removing the head and thorax, total RNA was isolated from the abdomen. RNA extraction was conducting utilizing the SteadyPure RNA Extraction Kit (Accurate Biotechnology (Hunan, China) Co., Ltd.), and total RNA quantification was performed using the NanoDrop OneC Micro UV–Vis spectrophotometer (Thermo Scientific, USA). Subsequently, RNA was reverse transcribed into cDNA for qRT-PCR analysis. The reaction system (20 μL) comprised the following: master mix (10 μL), 10 μM forward primer (0.5 μL), 10 μM reverse primer (0.5 μL), cDNA (500 ng), and ddH_2_O added to a final volume of 20 μL. The program settings were as follows: 95 °C for 180 s, followed by 45 cycles at 95 °C for 10 s, 59 °C for 30 s; 95 °C for 10 s, 65 °C for 60 s, 97 °C for 1 s. This method was utilized to determine alterations in the relative expression levels of reproductive-related genes in the FZYB group and FZY group from days 1 to 7 after mosquito emergence. The reproduction-related genes detected were: vitellogenin (*Vg1*, Gene ID: LOC120413209; *Vg3*, Gene ID: LOC120428119), vitellogenin receptor (*VgR*, Gene ID: LOC120420237), juvenile hormone receptor (*Met*, Gene ID: LOC120412890), ecdysone receptor (*EcR*, Gene ID: LOC120415091), and insulin-like peptide receptor (*ILPR*, Gene ID: LOC120418613). *β-actin* (Gene ID: LOC120432082) from *Cx. pipiens pallens* was used as the internal control (Table 1). The relative expression levels of each target gene in the FZY and FZYB groups were analyzed using the 2^−ΔΔ*Ct*^ method [35]. All experiments consisted of abdomens from 10 female mosquitoes, with three biological replicates per experiment.

In order to confirm that *B. aurantiaca* promotes mosquito reproduction via vitellogenesis, 20-hydroxyecdysone (20E), juvenile hormone (JH), and the concentrations of vitellogenin were measured in the FZYB group and the FZY group from days 1 to 4 after mosquito emergence. Five abdomens of female mosquitoes were used for each group, added 150 μL PBS buffer, thoroughly grinded, centrifuged at 5000 g for 10 min, and the supernatant was collected for detection. The 20E was detected using Insect 20-hydroxyecdysone (20-HE) ELISA Detection Kits (Enzyme-linked Biotechnology Co., Ltd. Shanghai, China), the JH was detected using Insect juvenile hormone I (JH-I) ELISA Detection Kits (Enzyme-linked Biotechnology Co., Ltd. Shanghai, China), and Insect VTG ELISA Detection Kit (Enzyme-linked Biotechnology Co., Ltd. Shanghai, China) were utilized to detect the concentrations of vitellogenin. The experimental procedures performed strictly in accordance with the manufacturer’s instructions, and all experimental procedures were performed with three replicates.

### Effects of the symbiotic bacterium *B. aurantiaca* on the autogeny of anautogenous mosquitoes

To investigate the modulatory impact of *B. aurantiaca* on the autogeny of anautogenous *Cx. pipiens pallens*, a continuous colonization approach across all developmental stages was adopted for bacterial treatment in this study. Specifically, the strain was continuously introduced to anautogenous mosquitoes at the egg, larval, pupal, and adult stages to ensure its stable colonization throughout the different developmental stages of the mosquitoes. The negative control group was a routinely reared population. During the experiment, egg rafts, larvae, pupae were maintained in sterile water containing a bacterial suspension concentration of approximately 3.6 × 10^8^ CFU/mL, fed conventionally and renewed every 2 days. The sucrose-soaked cotton balls with bacterial suspension were replaced daily for adult mosquitoes [18]. To assess the long-term effects, the bacteria-treated group was continuously reared up to the 5th generation. Additionally, life history traits were measured, including larval developmental duration, pupation rate, pupal duration, eclosion rate, and male-to-female sex ratio [36]. A blood meal was provided to adult mosquitoes at 3 days post-treatment, and reproduction-related parameters were recorded, including blood-feeding rate, oviposition rate, and hatching rate [37]. Larval life-history traits were measured with 30 larvae per group and three biological replicates. For reproductive indicators, adult mosquitoes were offered blood meals three days after eclosion. Oviposition performance was recorded over seven days. Each group contained 30–80 female mosquitoes across three biological replicates, and hatching rates were determined using ten egg rafts.

### Statistical analysis

This study used GraphPad Prism 10.1.2 software for statistical analysis and chart creation. For continuous variables such as gene relative expression levels, larval duration, and pupal duration, which followed a normal distribution, two independent sample *t*-tests were utilized for comparisons between groups. For variables that did not follow a normal distribution or had unequal variances, the Mann–Whitney test was utilized for comparisons between groups. For categorical variables such as pupation rate, emergence rate, sex ratio, blood-sucking rate, egg-laying rate, and hatching rate, the *χ*^2^ test was used for comparisons. All data are presented as means ± SDs. All statistical analyses were conducted at a significance level of *α* = 0.05.

## Results

### Morphological and molecular identification of the symbiotic bacterium *B. aurantiaca*

**Fig. 1:**
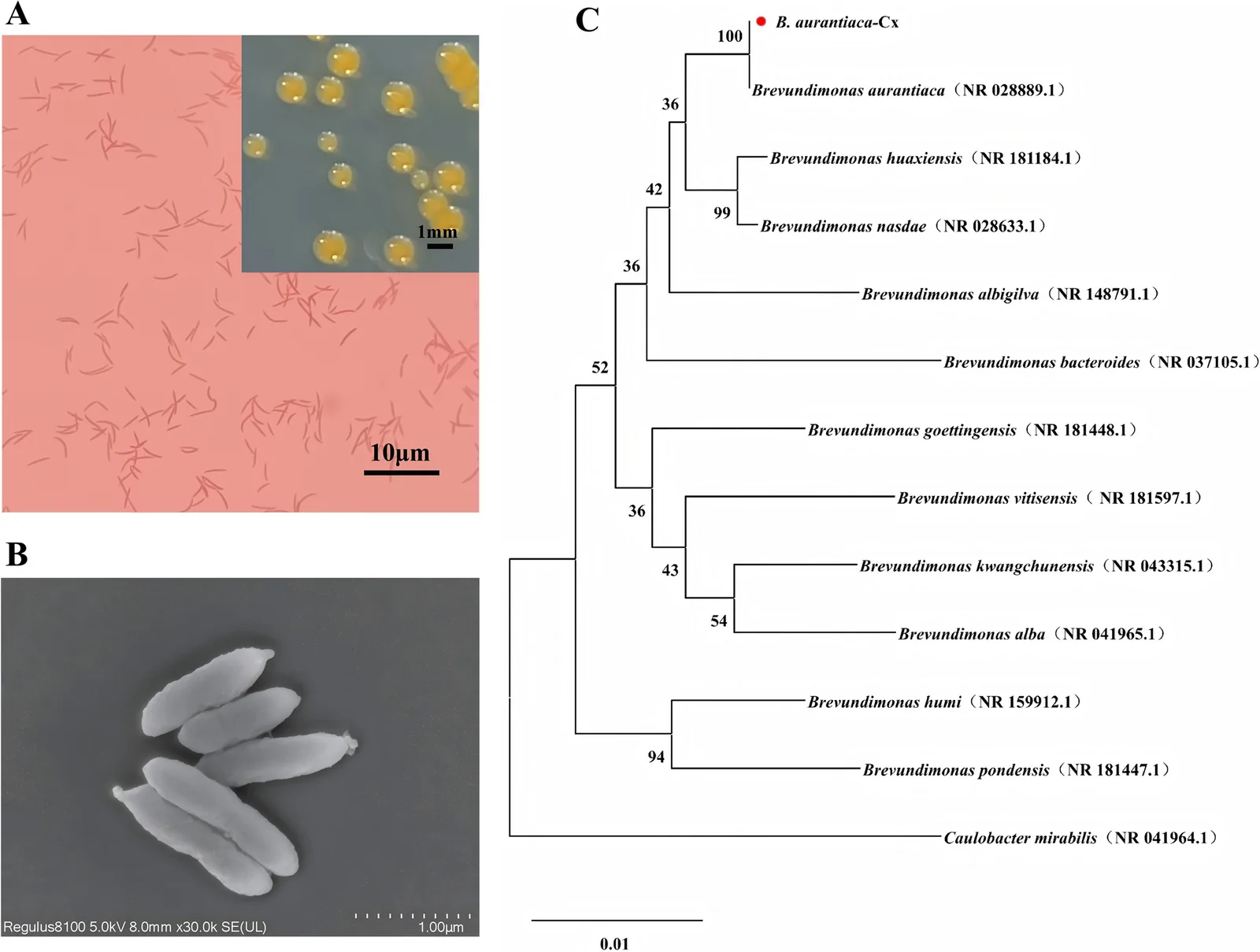
Morphological characteristics, Gram staining (**A**), scanning electron microscopy (SEM) (**B**), and phylogenetic tree (**C**) of the isolated strain

The bacterial strain isolated from autogenous *Cx. pipiens pallens* was identified as *B. aurantiaca* based on morphological, physiological, biochemical, and phylogenetic analyses. The detailed identification results are as follows. The isolated strain was a Gram-negative bacterium with red, rod-shaped cells of irregular arrangement. On CM agar plates, the bacterium formed orange-yellow, opaque, circular colonies with a convex, smooth, moist surface and an entire margin (Fig. 1A). Scanning electron microscopy (SEM) observations revealed that the isolated bacterial strain exhibited a typical rod-shaped morphology, mostly arranged in short chains with an intact and smooth surface. No flagella, pili, or other obvious surface appendages were observed, and strain had an approximate length of 1.0–1.5 μm and a diameter of 0.4–0.6 μm (Fig. 1B). 16S rRNA gene sequencing and BLAST analysis demonstrated that the amplified sequence of the strain had the highest similarity (99.6%) with that of *B. aurantiaca* strain (NR_028888.1). Phylogenetic tree analysis further revealed that the isolated strains clustered into a stable and independent branch with the members of the genus *Brevundimonas*, and they naturally clustered into the same evolutionary branch with *B. aurantiaca* (NR_028888.1) (bootstrap value = 100). This supports its taxonomic status as this species. Based on the above results, the isolated strain was named *B. aurantiaca*-Cx (Fig. 1C).

### Growth curve and physiological and biochemical identification of the symbiotic bacterium *B. aurantiaca*

**Fig. 2:**
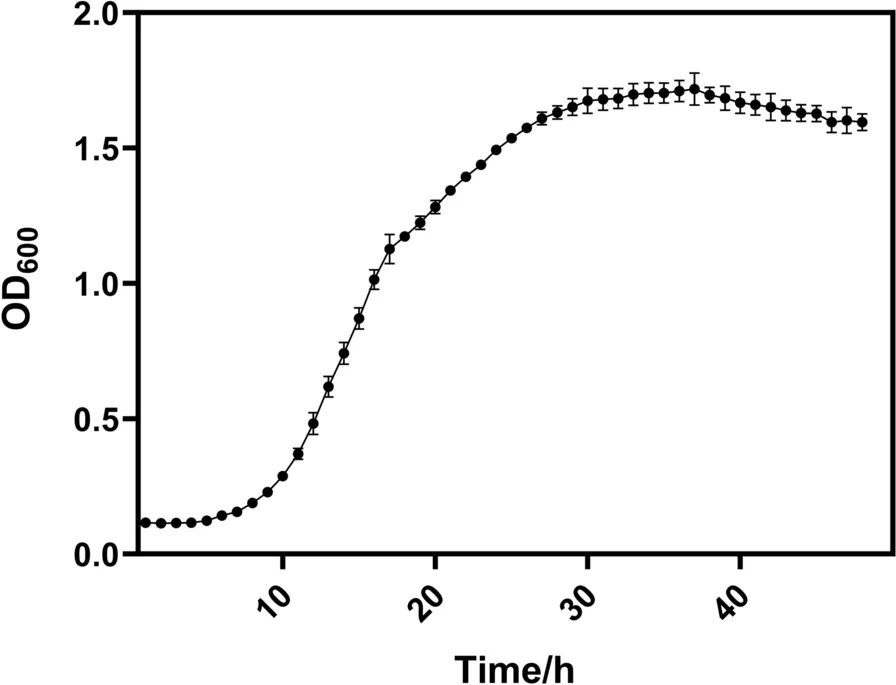
Growth curve of *B. aurantiaca* under liquid culture conditions. The bacterial strain was cultured in *Caulobacter* medium at 30 °C with continuous shaking at 180 rpm. Optical density at 600 nm (OD_600_) was monitored at set time points across a 48 h incubation period to characterize bacterial growth dynamics

The strain exhibited relatively slow growth, entering the logarithmic growth phase 10 h post-inoculation. The OD_600_ significantly increased from 0.3 to 1.7, and the proliferation rate reached the maximum. At 33–39 h after inoculation, the growth of the isolated strain plateaued, with the OD_600_ maintaining at approximately 1.7. The growth of the strain then entered the decline stage (Fig. 2). The isolated strain tested positive in the oxidase reaction and negative in the arginine dihydrolase, indole, nitrate reduction, glucose fermentation, and urease tests. It was unable to assimilate arabinose, mannose, mannitol, N-acetylglucosamine, gluconate, adipic acid, and phenylacetic acid, but it was able to assimilate glucose, maltose, capric acid, malic acid, and citric acid, and hydrolyze PNPG, gelatin, and aesculin. Based on the description of *Brevundimonas* in the Bergey's Manual of Systematic Bacteriology [38] and the aforementioned molecular biological identification results, the isolated strain was identified as *B. aurantiaca*.

### Dynamic distribution of *B. aurantiaca* during the development of autogenous *Cx. pipiens pallens* and its cross-strain colonization ability

**Fig. 3:**
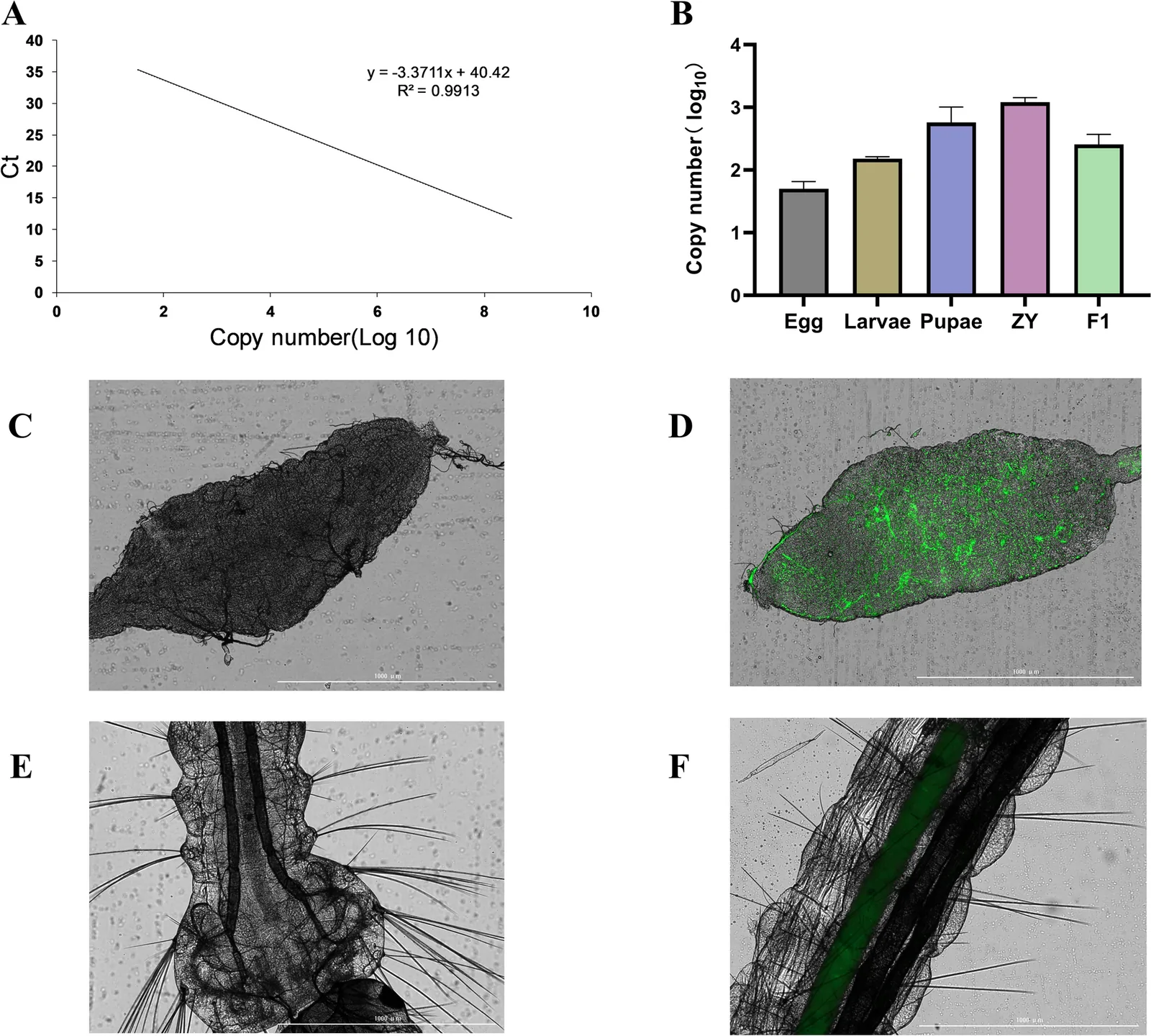
Colonization of fluorescently labeled *B. aurantiaca* in anautogenous *Cx. pipiens pallens* and its distribution across different developmental stages of autogenous *Cx. pipiens pallens*. **A** Absolute qRT-PCR standard curve. **B** Abundance of the bacterium (Log_10_ copy number) in egg rafts, larvae, pupae and newly emerged female adults of autogenous *Cx. pipiens pallens*, as well as in newly emerged female adults of the first generation from the bacteria-treated group (ZY represents autogenous female *Cx. pipiens pallens*, and F1 denotes newly emerged female adults of the first generation from the bacteria-treated group). **C** Midgut of female mosquitoes in control group. **D** Midgut of female mosquitoes in bacteria-treated experimental group. **E** 2nd-instar larvae in control group.** F** 2nd-instar larvae of the F1 progeny from the bacteria-treated experimental group

Absolute quantification by qRT-PCR, as an indirect approach to demonstrate bacterial colonization, revealed that *B. aurantiaca* was detectable at all developmental stages of autogenous *Cx. pipiens pallens*. The bacterial counts (Log_10_ copy number) in egg rafts, larvae, pupae, and newly emerged female mosquitoes were 1.7 ± 0.1, 2.2 ± 0.0, 2.8 ± 0.3, and 3.1 ± 0.1, respectively, with the highest abundance observed in newly emerged female mosquitoes. The bacterium was also detected in newly emerged female adults of the first generation from the bacteria-treated group (2.4 ± 0.2) (Fig. 3A, B).

Qualitative direct detection of GFP-tagged bacteria across the life phases showed that the plasmid expressing GFP was successfully introduced into *B. aurantiaca* by chemical transformation, GFP-labeled bacteria were visualized in the midgut of adult mosquitoes after feeding, confirming colonization (Fig. 3C, D). Furthermore, the detection of *B. aurantiaca* in F1 progeny suggests that the bacterium may be capable of vertical transmission (Fig. 3E, F).

### The expression levels of reproductive-related genes and the vitellogenesis assay in females

**Fig. 4:**
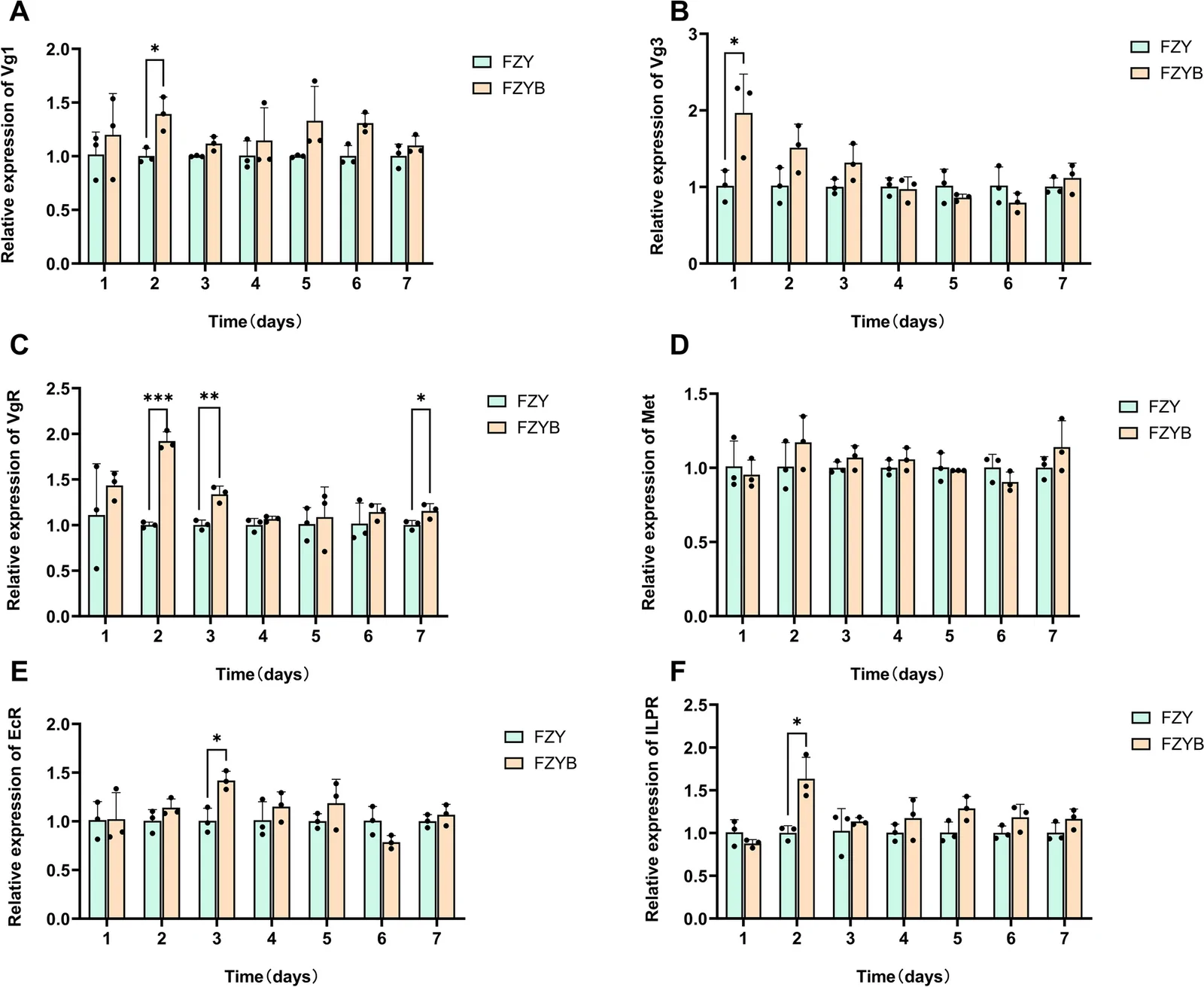
Expression levels of reproduction-related genes evaluated by qRT-PCR assays in the control group (FZY) and bacteria-treated group (FZYB) from day 1 to day 7 after mosquito emergence. A–F Represent the relative expression levels of *Vg1*, *Vg3*, *VgR*, *Met*, *EcR* and *ILPR* in the FZY and FZYB groups from day 1 to day 7 after mosquito emergence, respectively. *P < 0.05, **P < 0.01, and ***P < 0.001

Within 7 days of adult mosquito emergence, we systematically assessed the relative expression levels of the *Vg1*, *Vg3*, *VgR*, *Met*, *EcR*, and *ILPR* genes. qRT-PCR analysis found that the expression dynamics of major reproductive genes exhibited substantial alterations in the bacteria-colonized experimental group (FZYB) as compared with the non-inoculated control group (FZY) during the observation period (Fig. 4, raw data are shown in Supplementary material 1). Specifically, *Vg3* expression was significantly upregulated on day 1 (*t*-test, *t*_(4)_ =  − 3.008, *P* = 0.04), with levels markedly higher than those in the FZY group at the same time point. Although its expression fluctuated thereafter, *Vg3* remained elevated relative to the FZY group at most time points. *Vg1* expression was significantly upregulated on day 2 (*t*-test, *t*_(4)_ =  − 3.901, *P* = 0.018), and *VgR* expression was also significantly increased in the experimental group than in the control group on day 2 (*t*-test, *t*_(4)_ =  − 15.038, *P* < 0.001), indicating a synchronous enhancement in signal reception capacity during the peak period of vitellin synthesis.

Furthermore, the expression of the ecdysone receptor gene *EcR* in the FZYB group peaked on day 3, with it being significantly elevated as compared to the FZY group (*t*-test, *t*_(4)_ =  − 4.457, *P* = 0.011). Even though its expression gradually declined thereafter, *EcR* expression remained at a relatively high level, suggesting an enhanced sensitivity to 20-hydroxyecdysone (20E), the main hormone initiating vitellogenesis. In contrast, the expression of *Met*, the key mediator gene mediating the juvenile hormone JH signaling pathway, remained relatively stable. Although a slight increasing trend was observed in the FZYB group on day 2 and day 7, no statistically significant difference was observed between the two groups at any time point. Notably, the expression of the insulin-like peptide receptor gene *ILPR* was significantly enhanced in the FZYB group, with a significant increase detected on day 2 (t-test, *t*_(4)_ =  − 4.138, *P* = 0.014). This expression change coincided with the peak expression time of *VgR* and *Vg1*. These results inform that *B. aurantiaca* colonization was associated with an increase of expression reproduction-related genes in females.

**Fig. 5:**
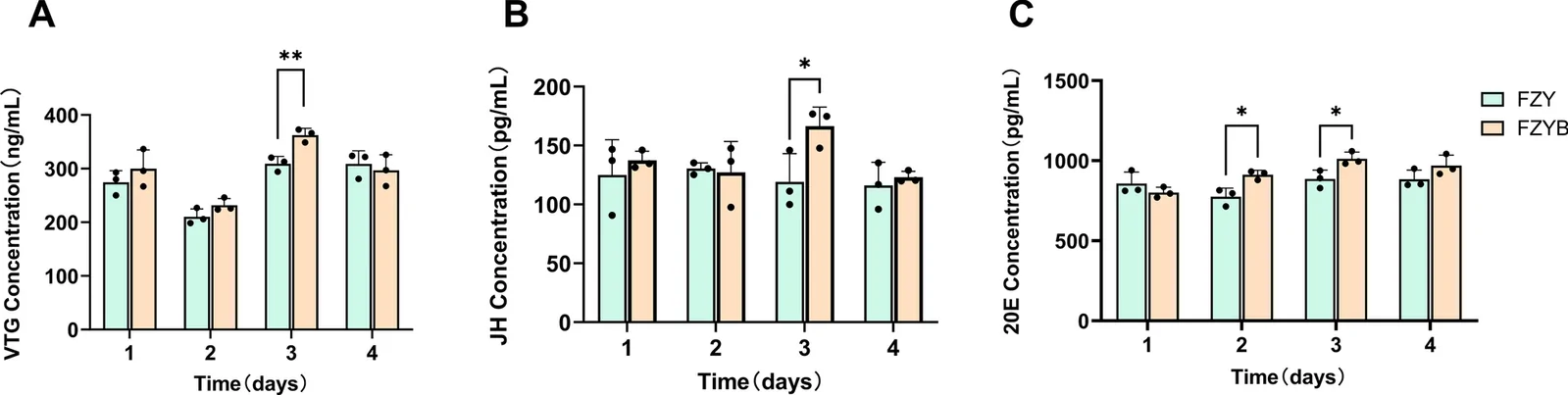
The vitellogenin protein, JH, and 20E levels determined by ELISA assays in the control group (FZY) and bacteria-treated group (FZYB) from day 1 to day 4 after mosquito emergence. A–C represent the vitellogenin protein, JH, and 20E levels in the FZY and FZYB groups from day 1 to day 4 after mosquito emergence, respectively. *P < 0.05, **P < 0.01

By measuring the levels of 20-hydroxyecdysone, juvenile hormone, and vitellogenin in the FZYB group and the FZY group from days 1 to 4 after mosquito emergence, the results showed an increasing trend in the concentrations of 20-hydroxyecdysone, juvenile hormone, and vitellogenin following bacterial treatment. 20-hydroxyecdysone increased significantly on the second (*t*-test, *t*_(4)_ = 3.888, *P* = 0.018) and third days(t-test, *t*_(4)_ = 3.186, *P* = 0.033). The JH levels increased significantly on the third day (t-test, *t*_(4)_ = 2.831, *P* = 0.047), and vitellogenin concentrations also showed a significant increase on the third day (*t*-test, *t*_(4)_ = 5.018, *P* = 0.007) (Fig. 5, raw data are shown in Supplementary material 2). The results suggest that *B. aurantiaca* may promote the vitellogenesis in mosquitoes by influencing the hormone levels of ecdysone and juvenile hormone, and the production of vitellogenin.

### Effects of the symbiotic bacterium *B. aurantiaca* on the fitness of anautogenous mosquitoes

Changes in the indicators of mosquito life history in the various treatment groups (anautogenous mosquitoes, bacteria-treated first generation, and bacteria-treated fifth generation) are shown in Table 2 (raw data are shown in Supplementary material 3). There was no significant difference in larval development time between the first generation of bacteria treatment and the control group, but the larval stage of the fifth generation was significantly prolonged (*t*-test, *t*_(4)_ =  − 4.186, *P* = 0.006), indicating that the delayed effect of bacteria on larval development gradually appeared after long-term passage. After *B. aurantiaca* treatment, the pupation rate and emergence rate of mosquitoes exhibited an upward trend, and remained stable in the fifth generation, indicating that *B. aurantiaca* treatment may affect the metamorphosis and development of mosquitoes. There was no substantial difference in the duration of pupal stage and the ratio of male to female between the treatment and control groups.

**Table 2:** Comparison of life history and reproductive traits among anautogenous, bacteria-treated F1, bacteria-treated F5 and autogenous mosquito groups

Traits		Anautogenous mosquito	Bacteria-treated F1	Bacteria-treated F5	Autogenous mosquito
Life history traits	Larval stage (d)	7.4 ± 0.2	7.2 ± 0.2	8.3 ± 0.4*	–
	Pupation rate (%)	86.7 ± 9.8	90.0 ± 5.5	92.5 ± 3.2	–
	Pupal stage (d)	1.4 ± 0.1	1.4 ± 0.0	1.5 ± 0.1	–
	Emergence rate (%)	87.6 ± 5.7	91.8 ± 5.4	91. 9 ± 3.5	–
	Male:female ratio	1: 1.2	1: 1.2	1: 1.3	–
Reproductive traits	Blood-feeding rate (%)	96.3 ± 2.7	91.8 ± 1.9*	87.7 ± 3.1**	–
	Oviposition rate (%)	48.2 ± 4.0	54.9 ± 3.8	58.7 ± 1.5	80.0 ± 7.0***
	Hatching rate (%)	97.7 ± 2.4	96.0 ± 1.5	97.0 ± 1.6	97.0 ± 1.7

* *P* < 0.05, **P < 0.01, ***P < 0.001 compared with anautogenous mosquito group

Regarding blood-feeding behavior, the control group had the highest blood-feeding rate. After *B. aurantiaca* treatment, the blood-feeding rate was significantly reduced, with the first generation of mosquitoes decreasing to 91.8% (Chi-square test, *χ*^*2*^ = 4.479, *df* = 1, *P* = 0.034) and the fifth generation further decreasing to 87.7% (Chi-square test, *χ*^*2*^ = 10.161, *df* = 1, *P* = 0.0014). In contrast, the rate of oviposition increased after *B. aurantiaca* treatment, but showed no significant difference compared with other groups, and there was also no substantial difference in the hatching rate of eggs among the three groups (Table 2), indicating that the bacteria had no observable impact on the fertilization and embryonic development of eggs.

## Discussion

Compared with the anautogenous mosquito species, the autogenous mosquitoes rapidly initiated the reproductive cycle after emergence without going through the host-seeking and blood-feeding risk period [3]. The primary difference between autogenous and anautogenous mosquitoes lies in the differentiation of reproductive and nutritional strategies, which is a systematic phenotypic feature of the synergistic effect of genetic regulation and environmental adaptation, and this differentiation demonstrates obvious differences in physiological metabolism, molecular regulation, and environmental adaptation [15, 39–41]. As a key symbiotic system in insects, the gut microbiota play an irreplaceable role in the modulation of insect growth and reproduction, which includes vitellogenesis, ovarian development, oviposition behavior and offspring survival, forming a co-evolution mechanism between host and microorganisms [42–46]. Specific intestinal bacteria can directly interfere with reproductive development through metabolic regulation or signaling pathways. For instance, *Rahnella aquatilis* hinders fecundity and ovarian maturation in female *Ae. albopictus* by decreasing the production of ecdysone and vitellogenin [21], whereas lactic acid bacteria in the gut of honey bee (*Apis cerana*) queens mediate ovarian metabolism via the purine metabolic pathway to drive ovarian activation [47]. Some intestinal bacteria can also indirectly enhance reproductive success by regulating host behaviors. For example, *Citrobacter* in the gut of the oriental fruit fly (*Bactrocera dorsalis*) produces 3-hexenyl acetate, which attracts hosts to oviposit at suitable sites, achieving precise matching between vertical bacterial transmission and host reproduction [48]. *Klebsiella* generates 2-ethylhexanol and 2,4-di-tert-butylphenol, which induce oviposition in gravid *Ae. aegypti* by mimicking oviposition signals [49].

In this study, we isolated and identified the gut-specific symbiotic bacterium *B. aurantiaca* from autogenous *Cx. pipiens pallens*. We found that the bacterium can stably colonize the autogenous *Cx. pipiens pallens* and that it possesses the ability for vertical transmission, exerting systemic effects on the growth, development and reproductive physiology of mosquitoes. Absolute qRT-PCR and green fluorescence observation confirmed that this bacterium could be consistently detected across all developmental stages of autogenous *Cx. pipiens pallens* and the bacterial counts increased in newly emerged female adults, indicating its successful adaptation to distinct microenvironments within the host, as well as its potential to proliferate during the adult stage and persist in mosquito populations. Colonization by *B. aurantiaca* was associated with the expression dynamics of key reproductive genes (*Vg*, *VgR*, *EcR*, *ILPR*) in mosquitoes. The expression of *Vg* and its receptor *VgR* serves as a central marker for reproductive initiation and oocyte maturation in female mosquitoes, and is precisely mediated by *Met*, *EcR*, and *ILPR* signaling pathways [50–52]. Our findings suggested that *B. aurantiaca* colonization correlated with higher transcript levels of *Vg1* and *VgR* in mosquitoes, suggesting that the bacterium may not only promotes vitellogenin synthesis but also synchronously enhances signal reception capacity. Additionally, the expression of *Vg3* peaked and remained at a high level at most subsequent time points. The significant upregulation of *Vg3* further indicates the global activation of the vitellogenin synthetic pathway may be possible. After bacterial treatment, the concentration of vitellogenin also increased significantly as measured. Collectively, these results suggest that colonization by *B. aurantiaca* may provide a more sufficient material basis for oocyte development by promoting the synthesis and transport of vitellogenin.

In terms of hormone signaling pathway, *B. aurantiaca* colonization was associated with the upregulated expression of *EcR*, which is a key regulator of ecdysone signaling, and the concentrations of 20-hydroxyecdysone showed a significant increase on the second and third days, suggesting that *B. aurantiaca* may promote the synthesis and secretion of 20E, a major hormone that initiates vitellogenesis. This finding is consistent with previous reports that bacteria can influence the host juvenile hormone-ecdysone axis [53, 54]. Most importantly, we observed that this bacterium could substantially increase the expression of *ILPR*, and the peak of *ILPR* expression coincided with the peak of *Vg1* and *VgR*. The insulin signaling pathway is the core hub linking nutritional status and reproduction [55, 56]. After emergence, the insulin signaling pathway is activated to initiate vitellogenesis, driving the massive synthesis of vitellogenin in the fat body and prioritizing reproductive development over other metabolic activities, such as flight muscle development or glycogen storage [4, 8, 15]. Gut microbiota may interact with host insulin/TOR signaling pathways via neurotransmitters such as serotonin, jointly regulating nutritional metabolism and reproductive development after blood feeding. However, the direct molecular mechanism remains to be experimentally verified [57, 58]. The results of this study suggest that *B. aurantiaca* may interfere with the nutrient sensing and insulin signaling pathway in the host and synergize with 20E signaling to drive the occurrence of vitellogenesis. In contrast, the expression of *Met*, a key gene in the juvenile hormone signaling pathway, was not significantly altered, however, the level of juvenile hormone has increased, indicating that *B. aurantiaca* influenced growth and reproduction in the mosquito in a pathway-specific manner.

Our results further confirm that the effects of *B. aurantiaca* on the developmental and reproductive phenotypes of *Cx. pipiens pallens* exhibit a transgenerational effect. Long-term exposure to *B. aurantiaca* gradually delayed the development of mosquito larvae by one day, since autogenous females had increased nutrient storage efficiency during the larval stage [4, 15], this result indicated that the bacterium may suppress the larval development and increase the nutritional and energy storage during the larval period. Continuous treatment by *B. aurantiaca* also promote the subsequent transformation process—that is, substantially decrease the blood-feeding rate of female mosquitoes (from 96.3 to 87.7%), but enhance the oviposition rate (48.2 to 58.7%). This effect was stable after passage, which was consistent with the developmental phenomenon of autogenous mosquitoes observed in our study. Combined with the observed changes in reproductive gene expression, our data suggest that *B. aurantiaca* colonization may influence host reproductive output under reduced blood-feeding conditions. Notably, significant alterations were observed in gene expression and hormone profiles, whereas changes in oviposition rate were not statistically significant. However, it is important to note that the current dataset establishes a correlative rather than causal relationship. The observed modulation of reproductive traits could involve multiple mechanisms, including bacterial metabolite supplementation, immune pathway regulation, or nutrient competition within the host, which remain to be functionally validated. Further refinement including the integration of additional experimental approaches and the exploration of other potential mechanisms is necessary to more conclusively establish the underlying regulatory pathways.

*B. aurantiaca*, as one of the gut-specific bacteria in autogenous *Cx. pipiens pallens*, has the greatest impact on the total yolk protein content of anautogenous mosquitoes according to our previous research (data not shown). As a vertically transmissible symbiotic bacterium, the interaction between *B. aurantiaca* and *Cx. pipiens pallens* may represent a microecological adaptation that lies between symbiosis and mild regulation, whereby the bacterium may modulates host reproduction-related pathways to influence the growth dynamics and reproductive potential of host populations. The long-term effects of this bacterium on mosquito populations are manifested in two specific aspects. First, it decreases the contact frequency between mosquitoes and humans via decreased blood-feeding rates, thereby directly lowering the risk of disease transmission. Second, it alters the mosquito life-history strategy, which in turn, alters the adaptive capacity and growth potential of the population in the environment. Therefore, the findings of this study provide a novel perspective for exploring microbe-based novel strategies for mosquito vector regulation.

## Conclusions

In this study, a commensal bacterium, *B. aurantiaca*, was isolated from the intestinal tract of autogenous *Cx. pipiens pallens* and characterized. It was demonstrated to stably colonize and achieve vertical transmission in anautogenous *Cx. pipiens pallens*. *B. aurantiaca* colonization was found to be associated with the upregulated expression of reproduction-related genes, which may contribute to alterations in the reproductive phenotype of the host. After several generations of treatment, the blood-feeding rate of anautogenous *Cx. pipiens pallens* decreased while the oviposition rate increased, suggesting that the bacterium may be associated with shifts in host reproductive strategy. However, whether *B. aurantiaca* directly promotes autogeny requires further investigation. Direct biochemical evidence is still required to confirm pathway activation. Future studies should establish a germ-free mosquito model, examine additional reproduction-related genes, and conduct field trials to further elucidate the bacterial role and assess its application potential.

## Supplementary Information

Additional file 1.

Additional file 2.

Additional file 3.

## Data Availability

All data supporting the findings of this study are available within the paper and its Supplementary Information.
